# Sonochemical synthesis and crystal structure of di­methyl­ammonium bis­[3-carb­oxy-2-(di­methyl­amino)­propano­ato-κ^2^ *N*,*O* ^1^]chlorido­chromium(II) monohydrate

**DOI:** 10.1107/S2056989019004717

**Published:** 2019-04-09

**Authors:** Meriem Saidi, Michel Giorgi, Leila Boukli-hacene

**Affiliations:** a Laboratoire de Chimie Inorganique et Environnement, Faculty of Science, University of Tlemcen, Tlemcen - 13000, Algeria; b Aix-Marseille University, Spectropole, Campus St. Jerome, 52 av. Escadrille Normandie Niemen, 13013 Marseille, France

**Keywords:** crystal structure, sonochemical synthesis, chromium(II) complex anion, di­methyl­ammonium, hydrogen bonding, supra­molecular framework

## Abstract

The title complex, [(CH_3_)_2_NH_2_][Cr(C_6_H_10_NO_4_)_2_Cl]·H_2_O, was synthesized sonochemically. The complex anion consists of a chromium(II) ion ligated by two 3-carb­oxy-2-(di­methyl­amino)­propano­ate anions. They coordinate in a bidentate manner, with a carboxyl­ate oxygen atom and the nitro­gen atom *cis* to each other in the equatorial plane, while the apical position is occupied by a Cl^−^ ion. Hence, the chromium(II) ion is five-coordinate with a quasi-ideal square-pyramidal geometry; τ_5_ parameter = 0.01. The complex crystallizes as a monohydrate and in the crystal, the water mol­ecule and the di­methyl­ammonium counter-ion link the complex cations *via* N—H⋯O, N—H⋯Cl, O_water_—H⋯O, O—H⋯O_water_ and O—H⋯O hydrogen bonds, forming a supra­molecular framework. There are also a number of C—H⋯O hydrogen bonds present that reinforce the framework structure. The crystal studied was refined as a racemic twin.

## Abstract 2

The title complex was synthesized sonochemically. The ligand was formed *in situ* by hydro­amination of fumaric acid. In the crystal, extensive hydrogen bonding, with the di­methyl­ammonium cation and the water mol­ecule linking the complex anions, results in the formation of a supra­molecular framework.

## Chemical context   

Fumaric acid, also known as *trans*-butenedioic acid, boletic acid, lichenic acid or allomaleic acid, occurs naturally in many plants and is named after *Fumaria officinalis*, a climbing annual plant (Felthouse *et al.*, 2001 ▸). Besides being ‘practically non-toxic’ (European Commission, 2003 ▸), it is used as an acidity regulator in the food industry (Linstrom & Mallard, 1998 ▸), in medicine (Gold *et al.*, 2012 ▸), and as a raw material in the manufacture of unsaturated polyester resins (Duty & Liu, 1980 ▸).

Since the beginning of the 21st century, fumaric acid has been used to synthesize one of the first metal–organic frameworks for commercial applications (Al-MOF: A520), presenting remarkable adsorption and mechanical properties, combined with low toxicity (Gaab *et al.*, 2012 ▸). In this context, the novel title compound was obtained during an attempt to synthesize a Cr–Fum MOF.
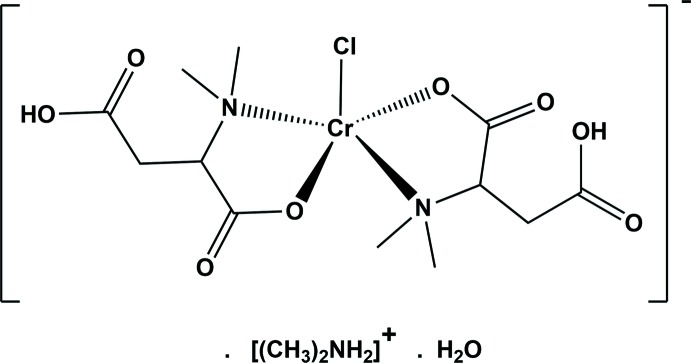


The reaction of fumaric acid and chromium(II)acetate dihydrate in the presence of di­methyl­amine hydro­chloride resulted in the hydro­amination of fumaric acid to form *N,N*-di­methyl­aspartic acid, which coordinates in a bidentate fashion to the chromium(II) ion.

## Structural commentary   

**Figure 1:**
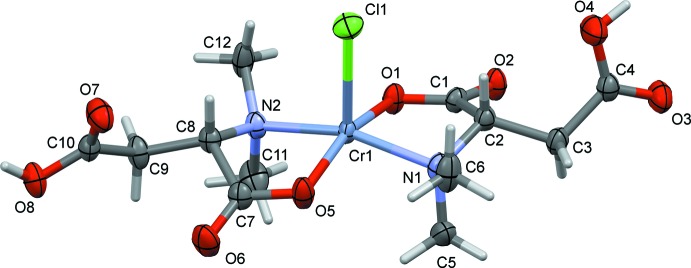
The mol­ecular structure of the title complex anion, with the atom labelling. Displacement ellipsoids are drawn at the 30% probability level. For clarity, the di­methyl­ammonium counter-ion and the water mol­ecule of crystallization have been omitted.

The mol­ecular structure of the title complex anion is illustrated in Fig. 1 ▸. The chromium(II) ion, atom Cr1, is coordinated to two 3-carb­oxy-2-(di­methyl­amino) propano­ate anions in a bidentate manner with a carboxyl­ate oxygen atom O1 and the nitro­gen N1 *cis* to each other for one ligand and for the other ligand atoms O5 and N2 are *cis* to each other. The chloride anion, Cl1, occupies the apical position. The five-coordinate chromium ion is displaced by 0.3469 (7) Å from the mean plane through atoms O1, N1, O5 and N2. The equatorial Cu—O bond lengths are Cr1—O1 = 1.960 (5) Å and Cr1—O5 = 1.954 (5) Å, while the equatorial Cu—N bond lengths are slightly longer *viz*. Cr1—N1 = 2.025 (5) Å and Cr1—N2 = 2.030 (5) Å. The axial Cr1—Cl1 bond length is 2.5301 (16) Å. The C—C, C—O, and C—N bond lengths of the ligands are close to those reported for similar compounds (Zheng *et al.*, 2003 ▸; Devereux *et al.*, 2000 ▸; Kim *et al.*, 2002 ▸). The *cisoid* and *transoid* bond angles vary from 83.62 (19) to 100.88 (16)° and from 159.6 (2) to 160.3 (2)°, respectively. This leads to a quasi-ideal square-pyramidal geometry for atom Cr1 with a τ_5_ parameter of 0.01 (τ_5_ = 0 for an ideal square-pyramidal geometry and 1 for an ideal trigonal–bipyramidal geometry; Addison *et al.*, 1984 ▸). An intra­molecular C6—H6*C*⋯O5 hydrogen bond (Table 1 ▸) occurs.

## Supra­molecular features   

**Figure 2:**
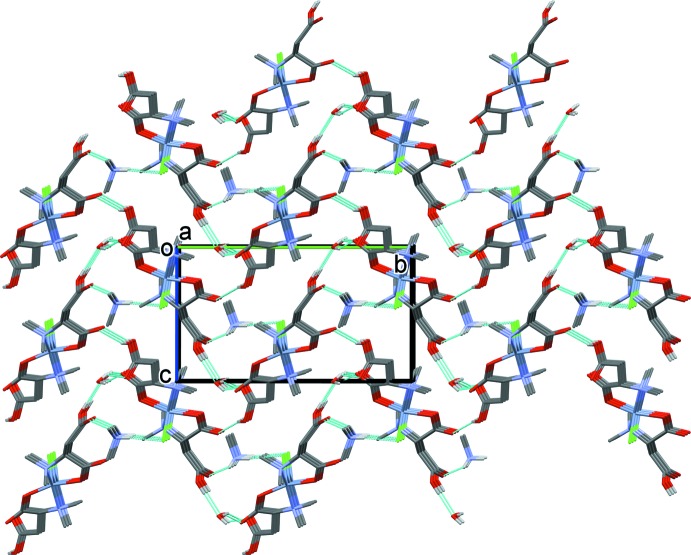
A view along the *a* axis of the crystal packing of the title complex. The hydrogen bonds (Table 1 ▸) are shown as dashed lines and, for clarity, all the C-bound H atoms have been omitted.

The crystal structure is stabilized by an extensive array of hydrogen bonds, forming a supra­molecular framework (Fig. 2 ▸ and Table 1 ▸). Beyond metal coordination, the ligand has potential sites for hydrogen bonding. Ten of the thirteen heteroatoms are involved in strong and moderate hydrogen bonds (Fig. 2 ▸ and Table 1 ▸). The complex crystallizes as a monohydrate and in the crystal, the water mol­ecule and the di­methyl­ammonium counter-ion link the complex cations *via* N—H⋯O, N—H⋯Cl, O_water_—H⋯O, O—H⋯O_water_ and O—H⋯O hydrogen bonds, forming a supra­molecular framework. There are also a number of C—H⋯O hydrogen bonds present that reinforce the framework structure.

## Database survey   

A search of the Cambridge Structural Database (CSD, Version 5.40, update February 2019; Groom *et al.*, 2016 ▸) indicated that there are no reports of chromium complexes of fumaric acid and no reports of the structure of the title ligand, *N*,*N*-di­methyl­aspartic acid. There is only one report of a complex containing a similar ligand, *viz*. [(*R*,*S*)-dimethyl 3-(di­phenyl­phosphino)-*N*,*N*-di­methyl­aspartate]di­chloro­palla­dium(II) [CASTIB; Chen *et al.*, 2012 ▸]. This chiral *P*,*N*-ligand was synthesized by hydro­phosphination using di­phenyl­phosphine followed by hydro­amination with a secondary amine.

## Synthesis and crystallization   

A mixture of fumaric acid (25 mg, 0.22 mmol) and di­methyl­amine hydro­chloride (0.09 ml) dissolved in 20 ml methanol was stirred for 1 h. Chromium(II) acetate dihydrate [Cr_2_(OAc)_4_·2H_2_O; 25.2 mg, 0.11 mmol] in 10 ml of water was added with magnetic stirring for a further 30 min. The mixture was then put in an ultrasonic bath (353 K, 45 KHz, 90 W) for 2h. The solution was then left to evaporate slowly and blue prismatic crystals were collected after two months.

## Refinement   

Crystal data, data collection and structure refinement details are summarized in Table 2 ▸. The crystal was refined as a racemic twin [BASF = 0.422 (11)]. The water H atoms were located in a difference-Fourier map and refined with a distance restraint of O—H = 0.85 (2) Å with *U*_iso_(H) = 1.5*U*_eq_(O). All other H atoms were placed in geometrically idealized positions and constrained to ride on their parent atoms: O—H = 0.82 Å, N—H = 0.89 Å, C—H = 0.96–0.99 Å with *U*_iso_(H) = 1.5*U*_eq_(O-hydroxyl, C-meth­yl) and 1.2*U*_eq_(N, C) for other H atoms.

## Supplementary Material

Crystal structure: contains datablock(s) I, Global. DOI: 10.1107/S2056989019004717/su5493sup1.cif

Structure factors: contains datablock(s) I. DOI: 10.1107/S2056989019004717/su5493Isup2.hkl

CCDC reference: 1892216

Additional supporting information:  crystallographic information; 3D view; checkCIF report

## Figures and Tables

**Table 1:** Hydrogen-bond geometry (Å, °)

*D*—H⋯*A*	*D*—H	H⋯*A*	*D*⋯*A*	*D*—H⋯*A*
O4—H4*O*⋯O1*W*	0.82	1.77	2.591 (8)	180
O8—H8*O*⋯O2^i^	0.82	1.91	2.585 (7)	139
N3—H3*C*⋯Cl1^ii^	0.89	2.24	3.121 (7)	172
N3—H3*D*⋯O3^iii^	0.89	1.94	2.763 (9)	153
O1*W*—H1*WA*⋯O7^ii^	0.86 (3)	2.17 (8)	2.895 (8)	142 (12)
O1*W*—H1*WB*⋯O6^iv^	0.86 (3)	2.18 (3)	3.006 (9)	160 (7)
C6—H6*B*⋯O8^v^	0.96	2.51	3.351 (9)	146
C6—H6*C*⋯O5	0.96	2.46	3.038 (9)	119
C12—H12*C*⋯O6^vi^	0.96	2.52	3.193 (10)	127
C13—H13*B*⋯O8^vi^	0.96	2.56	3.451 (12)	154

Symmetry codes: (i) 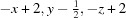; (ii) 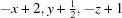; (iii) 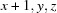; (iv) 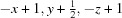; (v) 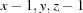; (vi) 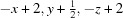.

**Table 2:** Experimental details

Crystal data	
Chemical formula	(C_2_H_8_N)[Cr(C_6_H_10_NO_4_)_2_Cl]·H_2_O
*M*_r_	471.86
Crystal system, space group	Monoclinic, *P*2_1_
Temperature (K)	298
*a*, *b*, *c* (Å)	8.2246 (2), 15.1419 (4), 8.6851 (2)
β (°)	93.339 (2)
*V* (Å^3^)	1079.77 (5)
*Z*	2
Radiation type	Cu *K*α
μ (mm^−1^)	5.94
Crystal size (mm)	0.16 × 0.10 × 0.06

Data collection	
Diffractometer	Rigaku Oxford Diffraction SuperNova, Dual, Cu at home/near, AtlasS2
Absorption correction	Multi-scan (*CrysAlis PRO*; Rigaku OD, 2018 ▸)
*T*_min_, *T*_max_	0.917, 1.000
No. of measured, independent and observed [*I* > 2σ(*I*)] reflections	11333, 3930, 3864
*R*_int_	0.039
(sin θ/λ)_max_ (Å^−1^)	0.605

Refinement	
*R*[*F* ^2^ > 2σ(*F* ^2^)], *wR*(*F* ^2^), *S*	0.053, 0.146, 1.08
No. of reflections	3930
No. of parameters	268
No. of restraints	4
H-atom treatment	H atoms treated by a mixture of independent and constrained refinement
Δρ_max_, Δρ_min_ (e Å^−3^)	1.14, −0.36
Absolute structure	Refined as an inversion twin
Absolute structure parameter	0.422 (11)

Computer programs: *CrysAlis PRO* (Rigaku OD, 2018 ▸), *SHELXT2014* (Sheldrick, 2015*a* ▸), *SHELXL2014* (Sheldrick, 2015*b* ▸), *Mercury* (Macrae *et al.*, 2008 ▸), *PLATON* (Spek, 2009 ▸), *OLEX2* (Dolomanov *et al.*, 2009 ▸), *PLATON* (Spek, 2009 ▸) and *publCIF* (Westrip, 2010 ▸).

## supplementary crystallographic information

### Crystal data

**Table:**

(C_2_H_8_N)[Cr(C_6_H_10_NO_4_)_2_Cl]·H_2_O	*F*(000) = 496
*M**_r_* = 471.86	*D*_x_ = 1.451 Mg m^−^^3^
Monoclinic, *P*2_1_	Cu *K*α radiation, λ = 1.54184 Å
*a* = 8.2246 (2) Å	Cell parameters from 7960 reflections
*b* = 15.1419 (4) Å	θ = 5.4–68.8°
*c* = 8.6851 (2) Å	µ = 5.94 mm^−^^1^
β = 93.339 (2)°	*T* = 298 K
*V* = 1079.77 (5) Å^3^	Prism, blue
*Z* = 2	0.16 × 0.10 × 0.06 mm

### Data collection

**Table:**

Rigaku Oxford Diffraction SuperNova, Dual, Cu at home/near, AtlasS2 diffractometer	3930 independent reflections
Radiation source: micro-focus sealed X-ray tube, SuperNova (Cu) X-ray Source	3864 reflections with *I* > 2σ(*I*)
Mirror monochromator	*R*_int_ = 0.039
Detector resolution: 5.3048 pixels mm^-1^	θ_max_ = 69.0°, θ_min_ = 5.1°
ω scans	*h* = −8→9
Absorption correction: multi-scan (CrysAlis PRO; Rigaku OD, 2018)	*k* = −18→18
*T*_min_ = 0.917, *T*_max_ = 1.000	*l* = −10→10
11333 measured reflections	

### Refinement

**Table:**

Refinement on *F*^2^	Secondary atom site location: difference Fourier map
Least-squares matrix: full	Hydrogen site location: mixed
*R*[*F*^2^ > 2σ(*F*^2^)] = 0.053	H atoms treated by a mixture of independent and constrained refinement
*wR*(*F*^2^) = 0.146	*w* = 1/[σ^2^(*F*_o_^2^) + (0.1071*P*)^2^ + 0.4201*P*] where *P* = (*F*_o_^2^ + 2*F*_c_^2^)/3
*S* = 1.08	(Δ/σ)_max_ < 0.001
3930 reflections	Δρ_max_ = 1.14 e Å^−^^3^
268 parameters	Δρ_min_ = −0.36 e Å^−^^3^
4 restraints	Absolute structure: Refined as an inversion twin
Primary atom site location: structure-invariant direct methods	Absolute structure parameter: 0.422 (11)

### Special details

**Table:**

Geometry. All esds (except the esd in the dihedral angle between two l.s. planes) are estimated using the full covariance matrix. The cell esds are taken into account individually in the estimation of esds in distances, angles and torsion angles; correlations between esds in cell parameters are only used when they are defined by crystal symmetry. An approximate (isotropic) treatment of cell esds is used for estimating esds involving l.s. planes.
Refinement. Refined as a 2-component inversion twin.

### Fractional atomic coordinates and isotropic or equivalent isotropic displacement parameters (Å^2^)

**Table:**

	*x*	*y*	*z*	*U*_iso_*/*U*_eq_	
Cr1	0.77728 (9)	0.46008 (6)	0.77478 (8)	0.0331 (3)	
Cl1	0.96891 (19)	0.44269 (11)	0.55923 (19)	0.0567 (4)	
O1	0.7158 (5)	0.5842 (3)	0.7439 (5)	0.0467 (10)	
O2	0.5397 (6)	0.6689 (3)	0.6095 (6)	0.0507 (10)	
O3	0.2468 (7)	0.6241 (5)	0.2902 (6)	0.0647 (16)	
O4	0.4927 (7)	0.5814 (5)	0.2465 (7)	0.0727 (16)	
H4O	0.473620	0.609797	0.167158	0.109*	
O5	0.7816 (6)	0.3408 (3)	0.8608 (6)	0.0568 (12)	
O6	0.8881 (8)	0.2659 (4)	1.0581 (7)	0.0679 (14)	
O7	1.2888 (7)	0.2838 (4)	1.0339 (6)	0.0686 (15)	
O8	1.2813 (7)	0.2989 (4)	1.2885 (6)	0.0578 (14)	
H8O	1.366024	0.270333	1.290499	0.087*	
N1	0.5641 (6)	0.4365 (3)	0.6532 (6)	0.0439 (11)	
N2	0.9374 (6)	0.4869 (3)	0.9555 (6)	0.0422 (11)	
C1	0.6020 (7)	0.5955 (4)	0.6425 (7)	0.0388 (12)	
C2	0.5452 (7)	0.5140 (4)	0.5487 (6)	0.0395 (12)	
H2	0.622057	0.505921	0.467737	0.047*	
C3	0.3768 (8)	0.5244 (5)	0.4683 (7)	0.0469 (13)	
H3A	0.337458	0.466321	0.437155	0.056*	
H3B	0.303802	0.547195	0.542577	0.056*	
C4	0.3661 (8)	0.5834 (4)	0.3295 (7)	0.0458 (13)	
C5	0.4376 (8)	0.4347 (6)	0.7677 (9)	0.062 (2)	
H8A	0.470785	0.395257	0.850250	0.093*	
H8B	0.336623	0.414570	0.718912	0.093*	
H8C	0.423522	0.492985	0.808217	0.093*	
C6	0.5626 (11)	0.3524 (5)	0.5675 (11)	0.067 (2)	
H6A	0.635292	0.356419	0.485227	0.100*	
H6B	0.454227	0.340600	0.525437	0.100*	
H6C	0.597207	0.305432	0.636040	0.100*	
C7	0.8884 (8)	0.3294 (4)	0.9699 (7)	0.0438 (12)	
C8	1.0189 (7)	0.4011 (4)	0.9867 (7)	0.0395 (12)	
H8	1.093246	0.391531	0.904066	0.047*	
C9	1.1207 (9)	0.3964 (4)	1.1380 (8)	0.0499 (14)	
H9A	1.181580	0.450948	1.151865	0.060*	
H9B	1.048333	0.391704	1.221919	0.060*	
C10	1.2384 (7)	0.3197 (4)	1.1473 (7)	0.0436 (12)	
C11	0.8385 (11)	0.5139 (6)	1.0840 (9)	0.067 (2)	
H11A	0.790995	0.570782	1.061941	0.100*	
H11B	0.906629	0.517222	1.177456	0.100*	
H19C	0.753661	0.471417	1.096277	0.100*	
C12	1.0545 (10)	0.5576 (5)	0.9208 (11)	0.067 (2)	
H12A	1.108472	0.542490	0.829352	0.100*	
H12B	1.133630	0.563742	1.005790	0.100*	
H12C	0.997048	0.612331	0.904808	0.100*	
N3	1.0276 (9)	0.7370 (4)	0.4178 (8)	0.0607 (15)	
H3C	1.036403	0.795574	0.417595	0.073*	
H3D	1.101606	0.715613	0.356926	0.073*	
C13	1.0657 (14)	0.7039 (7)	0.5796 (11)	0.079 (3)	
H13A	1.055159	0.640779	0.581505	0.119*	
H13B	0.991100	0.729742	0.647664	0.119*	
H13C	1.175078	0.720115	0.612551	0.119*	
C14	0.8635 (12)	0.7125 (7)	0.3526 (12)	0.078 (2)	
H14A	0.854076	0.727475	0.245084	0.118*	
H14B	0.782581	0.743983	0.406017	0.118*	
H14C	0.847500	0.650115	0.364519	0.118*	
O1W	0.4309 (7)	0.6707 (5)	−0.0045 (7)	0.0703 (15)	
H1WA	0.492 (14)	0.704 (8)	−0.056 (13)	0.105*	
H1WB	0.341 (10)	0.699 (8)	0.004 (8)	0.105*	

### Atomic displacement parameters (Å^2^)

**Table:**

	*U*^11^	*U*^22^	*U*^33^	*U*^12^	*U*^13^	*U*^23^
Cr1	0.0310 (4)	0.0267 (4)	0.0401 (4)	−0.0011 (3)	−0.0118 (3)	0.0011 (3)
Cl1	0.0540 (8)	0.0532 (10)	0.0636 (8)	−0.0009 (6)	0.0104 (6)	−0.0049 (6)
O1	0.049 (2)	0.033 (2)	0.056 (2)	−0.0003 (16)	−0.016 (2)	−0.0034 (17)
O2	0.053 (2)	0.033 (2)	0.064 (3)	0.0047 (18)	−0.017 (2)	0.0016 (19)
O3	0.057 (3)	0.079 (4)	0.057 (3)	0.025 (3)	−0.001 (2)	0.010 (3)
O4	0.058 (3)	0.094 (5)	0.067 (3)	0.023 (3)	0.008 (2)	0.022 (3)
O5	0.060 (3)	0.040 (2)	0.067 (3)	−0.009 (2)	−0.025 (2)	0.009 (2)
O6	0.082 (4)	0.047 (3)	0.072 (3)	−0.004 (2)	−0.015 (3)	0.019 (2)
O7	0.073 (3)	0.077 (4)	0.056 (3)	0.029 (3)	0.003 (2)	0.005 (3)
O8	0.071 (4)	0.048 (3)	0.052 (3)	0.016 (2)	−0.016 (2)	0.000 (2)
N1	0.040 (2)	0.035 (3)	0.055 (3)	0.0016 (18)	−0.011 (2)	0.002 (2)
N2	0.046 (3)	0.034 (3)	0.045 (2)	−0.0006 (19)	−0.009 (2)	0.0015 (18)
C1	0.038 (3)	0.033 (3)	0.045 (3)	−0.003 (2)	−0.005 (2)	−0.002 (2)
C2	0.037 (3)	0.038 (3)	0.043 (3)	−0.003 (2)	−0.006 (2)	0.000 (2)
C3	0.040 (3)	0.046 (3)	0.054 (3)	−0.007 (2)	−0.011 (2)	0.002 (3)
C4	0.046 (3)	0.041 (3)	0.049 (3)	−0.001 (3)	−0.010 (2)	−0.001 (3)
C5	0.044 (3)	0.071 (5)	0.071 (4)	−0.008 (3)	−0.003 (3)	0.026 (4)
C6	0.071 (5)	0.035 (4)	0.090 (6)	−0.001 (3)	−0.035 (4)	−0.008 (3)
C7	0.051 (3)	0.029 (3)	0.051 (3)	0.002 (2)	−0.005 (3)	−0.001 (2)
C8	0.044 (3)	0.030 (3)	0.044 (3)	0.004 (2)	−0.004 (2)	−0.002 (2)
C9	0.061 (4)	0.035 (3)	0.051 (3)	0.008 (3)	−0.016 (3)	−0.004 (2)
C10	0.042 (3)	0.039 (3)	0.048 (3)	0.001 (2)	−0.010 (2)	0.002 (2)
C11	0.080 (5)	0.061 (5)	0.059 (4)	0.027 (4)	0.001 (4)	−0.011 (3)
C12	0.063 (4)	0.046 (4)	0.086 (5)	−0.013 (3)	−0.039 (4)	0.013 (4)
N3	0.069 (4)	0.047 (3)	0.066 (3)	0.001 (3)	0.008 (3)	0.000 (3)
C13	0.101 (7)	0.066 (6)	0.072 (5)	0.011 (5)	0.010 (5)	0.003 (4)
C14	0.072 (5)	0.071 (6)	0.093 (6)	0.000 (4)	0.008 (5)	−0.014 (5)
O1W	0.063 (3)	0.070 (4)	0.080 (4)	0.001 (3)	0.013 (3)	0.019 (3)

### Geometric parameters (Å, º)

**Table:**

Cr1—O5	1.954 (5)	C5—H8C	0.9600
Cr1—O1	1.960 (5)	C6—H6A	0.9600
Cr1—N1	2.025 (5)	C6—H6B	0.9600
Cr1—N2	2.030 (5)	C6—H6C	0.9600
Cr1—Cl1	2.5301 (16)	C7—C8	1.527 (8)
O1—C1	1.259 (7)	C8—C9	1.518 (9)
O2—C1	1.250 (8)	C8—H8	0.9800
O3—C4	1.191 (8)	C9—C10	1.511 (9)
O4—C4	1.300 (9)	C9—H9A	0.9700
O4—H4O	0.8200	C9—H9B	0.9700
O5—C7	1.266 (8)	C11—H11A	0.9600
O6—C7	1.230 (8)	C11—H11B	0.9600
O7—C10	1.220 (9)	C11—H19C	0.9600
O8—C10	1.294 (8)	C12—H12A	0.9600
O8—H8O	0.8200	C12—H12B	0.9600
N1—C6	1.475 (9)	C12—H12C	0.9600
N1—C5	1.481 (9)	N3—C14	1.480 (12)
N1—C2	1.485 (8)	N3—C13	1.507 (11)
N2—C11	1.477 (9)	N3—H3C	0.8900
N2—C8	1.480 (7)	N3—H3D	0.8900
N2—C12	1.483 (9)	C13—H13A	0.9600
C1—C2	1.536 (8)	C13—H13B	0.9600
C2—C3	1.523 (8)	C13—H13C	0.9600
C2—H2	0.9800	C14—H14A	0.9600
C3—C4	1.499 (9)	C14—H14B	0.9600
C3—H3A	0.9700	C14—H14C	0.9600
C3—H3B	0.9700	O1W—H1WA	0.86 (3)
C5—H8A	0.9600	O1W—H1WB	0.86 (3)
C5—H8B	0.9600		
			
O5—Cr1—O1	159.6 (2)	N1—C6—H6C	109.5
O5—Cr1—N1	91.9 (2)	H6A—C6—H6C	109.5
O1—Cr1—N1	83.62 (19)	H6B—C6—H6C	109.5
O5—Cr1—N2	83.8 (2)	O6—C7—O5	123.1 (6)
O1—Cr1—N2	93.71 (19)	O6—C7—C8	121.5 (6)
N1—Cr1—N2	160.3 (2)	O5—C7—C8	115.3 (5)
O5—Cr1—Cl1	100.87 (19)	N2—C8—C9	114.9 (5)
O1—Cr1—Cl1	99.51 (15)	N2—C8—C7	107.3 (5)
N1—Cr1—Cl1	98.83 (16)	C9—C8—C7	113.5 (5)
N2—Cr1—Cl1	100.88 (16)	N2—C8—H8	106.9
C1—O1—Cr1	113.7 (4)	C9—C8—H8	106.9
C4—O4—H4O	109.5	C7—C8—H8	106.9
C7—O5—Cr1	114.1 (4)	C10—C9—C8	113.7 (5)
C10—O8—H8O	109.5	C10—C9—H9A	108.8
C6—N1—C5	109.7 (6)	C8—C9—H9A	108.8
C6—N1—C2	112.1 (6)	C10—C9—H9B	108.8
C5—N1—C2	111.9 (5)	C8—C9—H9B	108.8
C6—N1—Cr1	113.4 (4)	H9A—C9—H9B	107.7
C5—N1—Cr1	105.9 (4)	O7—C10—O8	124.7 (6)
C2—N1—Cr1	103.7 (3)	O7—C10—C9	123.1 (6)
C11—N2—C8	111.6 (5)	O8—C10—C9	112.1 (6)
C11—N2—C12	110.2 (7)	N2—C11—H11A	109.5
C8—N2—C12	112.3 (5)	N2—C11—H11B	109.5
C11—N2—Cr1	106.2 (5)	H11A—C11—H11B	109.5
C8—N2—Cr1	103.4 (4)	N2—C11—H19C	109.5
C12—N2—Cr1	112.8 (4)	H11A—C11—H19C	109.5
O2—C1—O1	124.0 (6)	H11B—C11—H19C	109.5
O2—C1—C2	119.0 (5)	N2—C12—H12A	109.5
O1—C1—C2	116.9 (5)	N2—C12—H12B	109.5
N1—C2—C3	115.0 (5)	H12A—C12—H12B	109.5
N1—C2—C1	107.0 (4)	N2—C12—H12C	109.5
C3—C2—C1	113.6 (5)	H12A—C12—H12C	109.5
N1—C2—H2	106.9	H12B—C12—H12C	109.5
C3—C2—H2	106.9	C14—N3—C13	114.1 (8)
C1—C2—H2	106.9	C14—N3—H3C	108.7
C4—C3—C2	116.1 (5)	C13—N3—H3C	108.7
C4—C3—H3A	108.3	C14—N3—H3D	108.7
C2—C3—H3A	108.3	C13—N3—H3D	108.7
C4—C3—H3B	108.3	H3C—N3—H3D	107.6
C2—C3—H3B	108.3	N3—C13—H13A	109.5
H3A—C3—H3B	107.4	N3—C13—H13B	109.5
O3—C4—O4	121.7 (7)	H13A—C13—H13B	109.5
O3—C4—C3	123.2 (6)	N3—C13—H13C	109.5
O4—C4—C3	114.9 (6)	H13A—C13—H13C	109.5
N1—C5—H8A	109.5	H13B—C13—H13C	109.5
N1—C5—H8B	109.5	N3—C14—H14A	109.5
H8A—C5—H8B	109.5	N3—C14—H14B	109.5
N1—C5—H8C	109.5	H14A—C14—H14B	109.5
H8A—C5—H8C	109.5	N3—C14—H14C	109.5
H8B—C5—H8C	109.5	H14A—C14—H14C	109.5
N1—C6—H6A	109.5	H14B—C14—H14C	109.5
N1—C6—H6B	109.5	H1WA—O1W—H1WB	107 (10)
H6A—C6—H6B	109.5		
			
Cr1—O1—C1—O2	176.8 (5)	Cr1—O5—C7—O6	164.7 (6)
Cr1—O1—C1—C2	−6.8 (7)	Cr1—O5—C7—C8	−14.7 (7)
C6—N1—C2—C3	70.4 (7)	C11—N2—C8—C9	−53.9 (8)
C5—N1—C2—C3	−53.3 (7)	C12—N2—C8—C9	70.4 (7)
Cr1—N1—C2—C3	−166.9 (4)	Cr1—N2—C8—C9	−167.7 (5)
C6—N1—C2—C1	−162.4 (6)	C11—N2—C8—C7	73.3 (7)
C5—N1—C2—C1	73.9 (6)	C12—N2—C8—C7	−162.4 (6)
Cr1—N1—C2—C1	−39.8 (5)	Cr1—N2—C8—C7	−40.5 (5)
O2—C1—C2—N1	−150.3 (5)	O6—C7—C8—N2	−140.5 (6)
O1—C1—C2—N1	33.1 (7)	O5—C7—C8—N2	38.9 (7)
O2—C1—C2—C3	−22.4 (8)	O6—C7—C8—C9	−12.5 (9)
O1—C1—C2—C3	161.1 (5)	O5—C7—C8—C9	166.9 (6)
N1—C2—C3—C4	−162.3 (5)	N2—C8—C9—C10	−163.7 (5)
C1—C2—C3—C4	74.0 (7)	C7—C8—C9—C10	72.3 (7)
C2—C3—C4—O3	−150.8 (7)	C8—C9—C10—O7	23.7 (10)
C2—C3—C4—O4	34.8 (9)	C8—C9—C10—O8	−157.9 (6)

### Hydrogen-bond geometry (Å, º)

**Table:**

*D*—H···*A*	*D*—H	H···*A*	*D*···*A*	*D*—H···*A*
O4—H4*O*···O1*W*	0.82	1.77	2.591 (8)	180
O8—H8*O*···O2^i^	0.82	1.91	2.585 (7)	139
N3—H3*C*···Cl1^ii^	0.89	2.24	3.121 (7)	172
N3—H3*D*···O3^iii^	0.89	1.94	2.763 (9)	153
O1*W*—H1*WA*···O7^ii^	0.86 (3)	2.17 (8)	2.895 (8)	142 (12)
O1*W*—H1*WB*···O6^iv^	0.86 (3)	2.18 (3)	3.006 (9)	160 (7)
C6—H6*B*···O8^v^	0.96	2.51	3.351 (9)	146
C6—H6*C*···O5	0.96	2.46	3.038 (9)	119
C12—H12*C*···O6^vi^	0.96	2.52	3.193 (10)	127
C13—H13*B*···O8^vi^	0.96	2.56	3.451 (12)	154

Symmetry codes: (i) −*x*+2, *y*−1/2, −*z*+2; (ii) −*x*+2, *y*+1/2, −*z*+1; (iii) *x*+1, *y*, *z*; (iv) −*x*+1, *y*+1/2, −*z*+1; (v) *x*−1, *y*, *z*−1; (vi) −*x*+2, *y*+1/2, −*z*+2.
